# The Undervalued Avenue to Reinstate Tumor Suppressor Functionality of the p53 Protein Family for Improved Cancer Therapy-Drug Repurposing

**DOI:** 10.3390/cancers12092717

**Published:** 2020-09-22

**Authors:** Joanna E. Zawacka-Pankau

**Affiliations:** Faculty of Chemistry, University of Warsaw, Pasteura 1, 02-093 Warsaw, Poland; j.zawackapan@uw.edu.pl or joannazawackapankau1@gmail.com

**Keywords:** p53, p73, MDM2, MDMX, tumor suppressor, drug repurposing, aspirin, protoporphyrin IX, verteporfin

## Abstract

**Simple Summary:**

Tackling the current dilemmas of cancer care, namely, financial and systemic burdens, is challenging. One way to address this challenge is to apply drug repurposing. Drug repurposing uses existing drugs for new medical indications like oncology. In drug repurposing, all clinical data is already in place, enabling fast translation into clinical applications. This review delineates the role of p53 and p73 as critical tumor suppressors and provides a comprehensive overview of drug repurposing avenues to reinstate the function of p53 proteins for cancer therapy.

**Abstract:**

p53 and p73 are critical tumor suppressors that are often inactivated in human cancers through various mechanisms. Owing to their high structural homology, the proteins have many joined functions and recognize the same set of genes involved in apoptosis and cell cycle regulation. p53 is known as the ‘guardian of the genome’ and together with p73 forms a barrier against cancer development and progression. The *TP*53 is mutated in more than 50% of all human cancers and the germline mutations in *TP*53 predispose to the early onset of multiple tumors in Li–Fraumeni syndrome (LFS), the inherited cancer predisposition. In cancers where *TP*53 gene is intact, p53 is degraded. Despite the ongoing efforts, the treatment of cancers remains challenging. This is due to late diagnoses, the toxicity of the current standard of care and marginal benefit of newly approved therapies. Presently, the endeavors focus on reactivating p53 exclusively, neglecting the potential of the restoration of p73 protein for cancer eradication. Taken that several small molecules reactivating p53 failed in clinical trials, there is a need to develop new treatments targeting p53 proteins in cancer. This review outlines the most advanced strategies to reactivate p53 and p73 and describes drug repurposing approaches for the efficient reinstatement of the p53 proteins for cancer therapy.

## 1. Introduction

It is the media hype and the unreasonable costs of the majority of new cancer treatments, often delivering only a marginal benefit, which harm cancer patients. More often than not, new treatments fail to deliver advancement in the outcomes, including overall survival. Surrogate endpoints applied in clinical trials usually include disease-free survival (DFS) (or progression-free survival), or overall response rates (ORR) as the primary outcome instead of a patient-centered, overall survival (OS). This, together with the underreported financial conflicts of interest among the decisive bodies, and the biased selection criteria for clinical trial randomization, all lead to the accelerated approval of expensive treatments which only marginally improve the patients’ outcome. As the situation looks now, it leaves little or no room for the introduction of the unbiased approach in cancer care, as illustrated by Vinayak Prasad in the book *Malignant* [1].

One way to overcome the burden of the skyrocketing costs of treatments of questionable benefit to patients is to apply a drug repurposing approach. Drug repurposing uses an existing drug for a different medical indication. In oncology, hard drug repurposing conveys the application of the drug from the non-oncology application, to improve the outcome of cancer therapy, often at a much lower cost than that of bringing a new treatment to the market [2]. This approach is economical as it takes advantage of the clinical information that is already available for the given drug, such as pharmacokinetic and pharmacodynamic profiles, maximum tolerated dose or clinical safety profile, which allows for shorter times for the treatment’s implementation into practice [3].

p53 and its ancestor family members, p73 and p63, evolved in the multicellular organisms as the sensors of the DNA damage. All proteins constitute a critical barrier against cancer development in humans and regulate the expression of genes involved in apoptosis, cell cycle regulation or DNA repair. p53 is the most commonly inactivated protein in human cancers, either due to the mutations in its gene promoting the loss of wild-type (wt) p53 function, or due to the overactivated oncogenic inhibitors, like MDM2 and/or MDMX [4]. p53 works together with p73, a p53 protein family member, which also includes p63. p73 evolved earlier than p53 in vertebrates and all three proteins share a similar sequence, architecture, and function. The structure–function similarity among the p53 protein family allows us to assume that small molecules activating p53 will also work on p73, which is discussed in Section 2 and Section 3.

In Li–Fraumeni syndrome, the inherited cancer predisposition, *TP*53 mutations have high penetrance, and the loss of p53 function drives the early onset of multiple tumors. The germline *TP*53 mutations make the treatment of LFS patients challenging due to the genotoxicity of currently available therapies, enhancing the probability of the development of secondary malignancies. Thus, the LFS patients are in majority treated with surgery before implementing chemotherapy or radiotherapy. Some hopes for improved therapy of tumors in LFS patients are seen with immunotherapies [5]. However, the cost of immunotherapies and other non-genotoxic modalities e.g., the CAR-T therapy (app. USD 2 million with accompanying costs), calls for urgent development of new, more affordable treatments for cancer patients with the mutated p53 [6]. 

Taken the abovementioned issues, the critical role of p53 in cancer initiation and progression, and the recently reported failure of the promising MDM2 inhibitors, RG7112, and idasanutlin in clinical trials, there is a need for enhanced efforts into development of therapies reactivating the p53 protein family [7]. This review describes structures and tumor suppressor functions of p53 and p73, selected approaches to reactivate p53 proteins’ function in tumors and highlights the potential of drug repurposing approach for restoration of p53 and p73 for cancer therapy.

## 2. Structure and Tumor Suppressor Function of p53 and p73

### 2.1. p53

**Figure 1:**
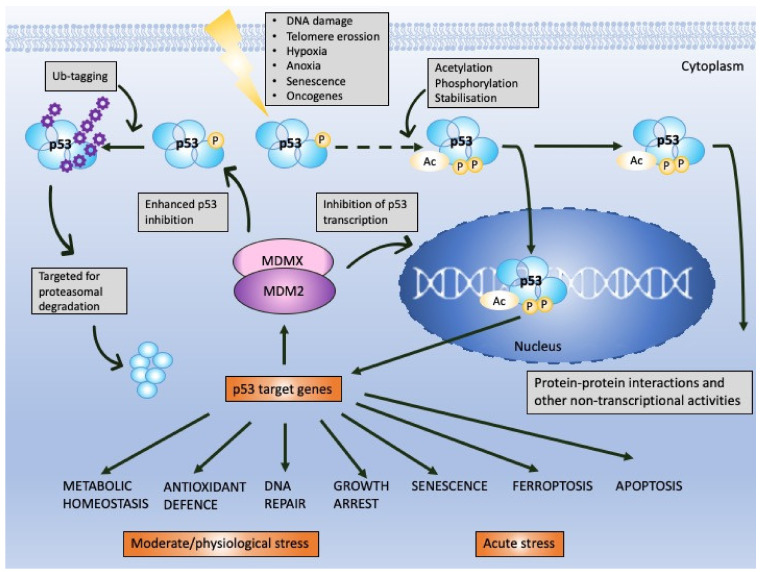
p53 and MDM2 as a hub of p53-dependent cellular responses—a simplified model. Under physiological conditions, p53 is degraded by MDM2, E3 ubiquitin ligase, which, depending on the level of cellular stress, can have either high or low affinity to p53. MDM2 is responsible for p53 monoubiquitination (driving p53 nuclear export) and polyubiquitination of p53 (driving p53 ubiquitin-dependent proteasomal degradation) and prevents p53 acetylation and transcriptional activation by p300 acetyltransferase. The affinity of MDM2 to p53 is enhanced upon hetero-dimerization with its homolog, MDMX protein. Upon stress, p53 undergoes phosphorylation and acetylation (the sites depend on the type and severity of stress) and recognizes its target genes. MDM2 and MDMX may prevent p53 from initiating the transcription through direct binding, which hinders the binding of the transcriptional co-activators. The sets of the target genes that become activated/repressed by p53 are often interrelated. In addition to transcription-dependent activity, cytoplasmic p53 functions through protein–protein interactions to modulate apoptosis, miRNA maturation or the repair of double-strand breaks (DBS). The dotted line represents a multistep process. Adapted from Levine, 2020 [13], Levine and Oren, 2009 [14] and Bode and Dong, 2004 [15].

p53 is a protein of the domain structure and a transcription factor binding specifically to DNA consensus sequence consisting of two consecutive half-sites as a tetramer [8]. p53 is known to undergo multiple post-translational modifications including phosphorylation, ubiquitination, sumoylation, neddylation, acetylation, methylation, or recently described UFMylation [9], which are necessary for p53 cellular turnover. In non-stressed cells, the half-life of p53 is around 20 min and the protein becomes stabilized and activated by the cascade of events provoked by cellular stress signals (reviewed in [10]). Stabilization of p53 is achieved by the decrease in the affinity of MDM2 to p53 (or HDM2 in humans), a major p53 E3 ubiquitin ligase which drives p53 for proteasomal degradation, in the cytosol and in the nucleus [11,12]. Activation of p53 transcription function occurs upon the inhibition of the binding of MDM2 to the N-terminal domain of p53 at the target DNA sequence. Since MDM2 is also a p53 target gene, a negative feedback loop exists that regulates p53 activity (Figure 1) [11]. 

Next, MDM2 protein, at mild stress, monoubiqutinates p53, enforcing its nuclear export and enabling p53 non-transcriptional activity. MDM2 activity towards p53 is enhanced by its homolog, MDMX protein, which lacks the E3 ligase activity but binds to the N-terminus of p53 and MDM2 alike, inhibiting its transcription function [16]. Apart from MDM2, other ligases play a role in altering p53 stability like Trim family members or Pirh or bacterial or viral proteins, such as SV40 or E6 protein of the HPV virus [17]. It is, however, the MDM2–p53 hub that is responsible for regulating multiple cellular processes in human cells, such as apoptosis, cell cycle, DNA repair, antioxidant response or senescence as well as metabolism (Figure 1) [13]. Furthermore, p53 also regulates ferroptosis, iron-related cell death and has the transcription-independent function in apoptosis (binding to Bcl2-family of proteins), in miRNA maturation (binding to Drosha-complex proteins) and in DNA repair [18]. Its pivotal role is to orchestrate the response to genotoxic, oxidative, and oncogene-induced stress [19]. 

In response to mild DNA damage, activation of p53 transcription initiates cell cycle inhibition, necessary for the DNA repair to occur, and both processes converge on a cascade of protein–protein interactions (PPIs) [13,20,21]. If the DNA damage cannot be repaired, the cell is directed to apoptosis, a programmed cell death. In that case, p53 transactivates BCL2-associated X, apoptosis regulator (*BAX*), p53 upregulated modulator of apoptosis (*PUMA*; also known as *BBC*3) and *NOXA* (also known as *PMAIP*1) [22] or interacts directly with the multidomain anti-apoptotic (Bcl-xL and Bcl-2) and proapoptotic (Bak) Bcl-2 members at mitochondria and induces mitochondrial outer membrane permeabilization and consequent cytochrome c release and apoptosis (reviewed in [23]). 

#### 2.1.1. p53 Structure

**Figure 2:**
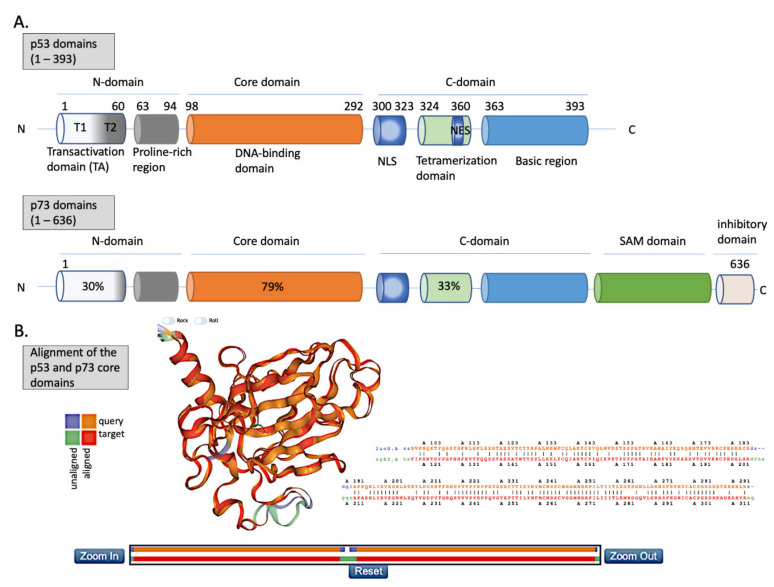
The structures of p53 and p73. (**A**) Upper panel—domains in p53 protein. Lower panel—percentage homology of residues between p53 and p73 are presented and the values are indicated for each individual structural domain. T1, T2—transactivation domain (TAD) 1 and 2; NLS—nuclear localization signal; NES—nuclear export signal; SAM—sterile alpha-motif. Adapted from Tanaka et al. [32], Joanna Zawacka-Pankau et al. [33] and Melino et al., [34]. (**B**) Structure alignment of p53 core domain (PDB ID 2AC0 [8]) and p73 core domain (PDB ID 4G82 [35]) generated using Top Match Services with opacity of unmatched pairs of 0.7. https://topmatch.services.came.sbg.ac.at/index_ngl.html [36]. At the C-terminus, the regulatory basic domain is located [37] which is involved in the interactions with DNA through non-specific DNA binding allowing for distinctive target gene recognition by p53 and p73 [38].

The N-terminus domain of p53 includes transactivation domain 1 (TAD1, depicted as T1) and TAD2 (T2) (Figure 2A, upper panel). TAD1 and 2 work synergistically to induce transcription, and are sites of phosphorylation events leading to inhibition of MDM2-p53 complex and to activation of p53-dependent response (reviewed in [24]). The X-ray structure of the MDM2 N-terminus and p53 N-terminal peptide complex shows that the minimal requirements for p53 to bind MDM2 are residues F^19^S^20^D^21^L^22^W^23^K^24^L^25^L^26^ [25,26]. p53 residues F19, W23 and L26 are responsible for binding with MDM2 and MDMX [27]. Taking into account the well-known structure of the MDM2-p53 complex, and the fact that the inhibition of the wild-type (wt) p53 via p53/MDM2/MDMX axis is essential for cancer to develop (reviewed in [4]), inhibition of the MDM2-p53 and MDMX-p53 interactions has become a very promising strategy for cancer therapy and is described in more detail below. The TAD domain of p53 is rendered unfolded and adapts a transiently stable secondary structure. In particular, the region from Phe19 to Leu22, responsible for binding to MDM2 protein, exhibits local helix propensity [28] and is sensitive to the charge-induced shifts. Interestingly, the liable p53 N-terminus can be targeted with small molecules that move the local charge and disrupt the helix. This prevents MDM2-p53 interactions as demonstrated for a small molecule RITA, a compound that affects the interaction between p53 and MDM2 through the change in conformation of p53 N-terminus [29,30]. Since this phenomenon is not fully understood yet, it will not be discussed in this review. 

p53 binds specifically to its consensus DNA sequence through the DNA binding domain. The DNA binding domain (DBD) located centrally, spans the amino acids from 98 to 292, is preceded with the proline-rich region and two transactivation domains, TAD1 and TAD2 (Figure 2A, upper panel). The DBD domain is enriched in cysteine residues and contains an antiparallel β-sheet sandwich supported by loops L1, L2, and L3. Loops L2 and L3 contain amino acids for a tetrahedrally coordinated Zn^2+^ ion. The wild-type p53 protein recognizes the canonical DNA sequence motif by binding to DNA through residues K120, R273, A276, C277, R280, R283 and residues S241 and R248, which are located at the ends of two β-sheets [31]. Since the DBD is important for p53-facilitated transcription, it is a site of multiple inactivating mutations which are found in cancer. 

#### 2.1.2. p53 Inactivation in Cancer

p53 is activated in response to oncogene-induced stress (Figure 1) and is therefore the most commonly mutated gene in cancer. More than 50% all of human cancers harbor the inactivating mutations and the six most common are the missense mutations hindering the activity of DBD domain: R175, G245, R248, R249, R273, R282 ([39,40] https://p53.iarc.fr/). The mutations render p53 inactive and/or promote the gain of new functions [41]. Studies demonstrated the feasibility of reactivating mutant p53 with small molecules (reviewed in [42]) and an advanced clinical example is described below. In cases in which *TP*53 gene remains intact, p53 protein is degraded by the upregulated or hyperactive MDM2 protein, which acts in concert with MDMX (reviewed in [4,13]) (Figure 1). MDM2 was found to be overexpressed in many tumor types via several mechanism including gene amplification or enhanced transcription [43]. *MDM*2 is amplified in sarcomas, bladder cancer or glioblastoma (https://www.cbioportal.org/). The protein is expressed from two promoters [44] and the single nucleotide polymorphisms (SNP), SNP309G-allele and SNP55T-allele in promoter 2 of MDM2 were described to enhance the binding of Sp1 transcription factor and to increase MDM2 expression. Accumulated MDM2 promotes p53 downregulation in several human cancers [45,46,47]. Next, MDM2 is overactivated in cancers because of the inhibition of p14ARF tumor suppressor. In normal cells, oncogene activation stimulates p53 stabilization due to activation of p14ARF. p14ARF binds to MDM2 and induces the nucleolar import of MDM2 protein. The binding of p14ARF prevents MDM2-mediated transactivational silencing of p53 and p53 degradation [48,49]. p14ARF was reported to be inhibited in cancer cell lines and tumor tissues through the INK4a/ARF locus deletion or promoter hypermethylation, and the homozygous deletions of p14ARF have prognostic significance in cancer [50,51,52]. Similarly to ARF, MDM2 is also negatively regulated by the ribosomal proteins (RP) L11 and RPL5 [53] that are activated by ribosomal or nucleor stress. 

The growing evidence implies that the mechanisms leading to p53 inactivation in cancer, to some extent, also apply to p73, a p53 protein family member. For example, p73 is activated by RBL11 and RBL5 in cancers [54]. This will be discussed in more detail in Section 2.2.

#### 2.1.3. Pharmacological Reactivation of p53

The most advanced mutant p53 reactivating compound is APR-246 (known as eprenetapopt) discovered by Klas Wiman and colleagues [55]. APR-246 is converted to methyl quinuclidinone (MQ) and acts as Michael acceptor which targets specific cysteine residues in p53 core domain [56,57]. The binding of MQ to cysteine 277 increases the thermostability of the core domain in vitro and cysteine 124 and 277 are crucial for reactivation of mutp53-R175H in cancer cells. APR-246 also inhibits thioredoxin reductase (TrxR) and binds to gluthatione which boosts accumulation of reactive oxygen species and contributes to cancer cells’ death (reviewed in [58]). The compound is studied in Phase III clinical trial in combination with azacytidine in *TP*53 mutated myelodysplastic syndrome (MDS) and acute myeloid leukemia (AML) (reviewed in [4,59]).

Extensive studies led to the development of rationally designed small-molecule inhibitors, nutlins, that bind the MDM2 hydrophobic pocket with high affinity, and efficiently outcompete p53 from the binding site [60]. The pivotal study with nutlin-3 (IUPAC: 4-[(4S,5R)-4,5-bis(4-chlorophenyl)-2-(4-methoxy-2-propan-2-yloxyphenyl)-4,5-dihydroimidazole-1-carbonyl]piperazin-2-one), showed that it mimics the three key interactions of p53. Specifically, the imidazoline fits into the MDM2 binding site protruding three hydrophobic groups into subpockets that are normally occupied by the p53 Phe19, Trp23, and Leu26 residues and the piperazine ring attached to the N1 of the imidazoline is outside the binding site and does not contact MDM2. Nutlin has a much lower affinity to MDMX [61,62] and thus, is ineffective in tumors that overexpress both MDM2 and MDMX [63]. Similarly to MDM2, p53 regulates MDMX as it binds to mRNA of MDMX and regulates its translation. More specifically, the p53 DBD domain binds the 5′ untranslated region (UTR) of the MDMX mRNA in a zinc-dependent manner and through the partaking of the p53 N-terminus controls MDMX synthesis generating a negative feedback loop between p53 and MDMX as is the case for MDM2 [64]. The initial success of nutlin-3 commenced the development of a series of potent MDM2-p53 inhibitors and their extensive testing in the clinical setting. However, recently the failure of highly specific MDM2 inhibitors, RG7112 and idasanutlin in clinical trials was reported [7]. Yet, a compound called APG-115, an oral MDM2 inhibitor of high affinity, was tested in combination with KEYTRUDA^®^ in a Phase Ib/II trial [65]. Further studies will show the clinical efficacy of the drug.

One of the promising strategies to treat cancers with wtp53 is to apply dual inhibitors of MDM2-p53 and MDMX-p53 interactions [63]. The most advanced examples of such approach are stapled peptides, α-helical p53 stapled peptidomimetics among which the ALRN-6924 peptide is the only one in early phase clinical development [66]. Small-molecule, dual antagonists have not yet been tested in the clinical setting and thus, new approaches allowing for rapid translation into clinical practice are needed.

An emerging strategy to target tumors with inactive p53 is to reactivate other p53 protein family members. p73 is an important tumor suppressor, rarely mutated in cancer. The accumulated published data imply that p73, when reactivated, compensates for p53 loss and induces apoptosis and tumor regression in vivo, as discussed in detail below.

### 2.2. p73

Since its discovery in 1997, p73 has been intensively studied because of its high structural similarity to p53 and owning to the possibility to compensate for p53 loss in tumors [67]. p73 has higher than p63 percentage of the homology in the DNA binding domain to p53 (Figure 2A,B (lower panel)) and forms open tetramers in a manner similar to p53, while p63 forms two closed dimers [68,69]. Such similarity to p53 allows to make an assumption that p73 might recognize and activate many of p53 target genes and that similar pharmacological approaches can be employed to activate p73 protein for cancer therapy. Taking into consideration the difference in structure and the limited data regarding p63 reactivation for cancer therapy, this review will focus on p53 and p73 solely.

#### 2.2.1. p73 Structure

p73 is expressed in several isoforms that have distinct functions. The two major p73 isoforms dictating the cell fate upon cellular stress and chemotherapy treatment are TA isoforms and ΔN isoforms. p73 has two promoters—P1 in the 5′ untranslated region upstream of the noncoding exon 1, and P2 within the 23 kb spanning intron 3, triggering the synthesis of two distinct isoforms (reviewed in [70]). TA isoforms are transcriptionally active and act as tumor suppressors and ΔN isoforms, which lack the N-terminus, arise in cells through the alternative promoter usage of P2 and through the alternative splicing. Importantly, when the ratio between the isoforms is altered due to, e.g., the methylation of CpG islands in promoter 1, ΔN isoforms accumulate and can interact with and inhibit TA isoforms and p53 [71]. In addition to inhibiting p53 and p73, ΔNp73 has other oncogenic functions such as binding to HIF1a and promoting its stability and tumor metastasis [72], driving chemoresistance by regulating the expression of the multi-drug resistance genes ABCB1 and 5 [73], interacting with TGFβ signaling by inducing its target genes *PAI*-1 and *Col*1a1 [74], or inhibiting PTEN tumor suppressor [75,76]. Next, the alternative splicing at the C-terminus generates the C-terminal isomeric forms of p73, which are expressed both in healthy and in cancer cells. The longest isoform, TAp73α, contains a highly conserved sterile motif (SAM) (Figure 2A (lower panel)), which is a protein–protein interaction module (reviewed in [34]). In total, there are 35 isoforms of p73, which adds complexity while studying p73 biology [77].

**Figure 3:**
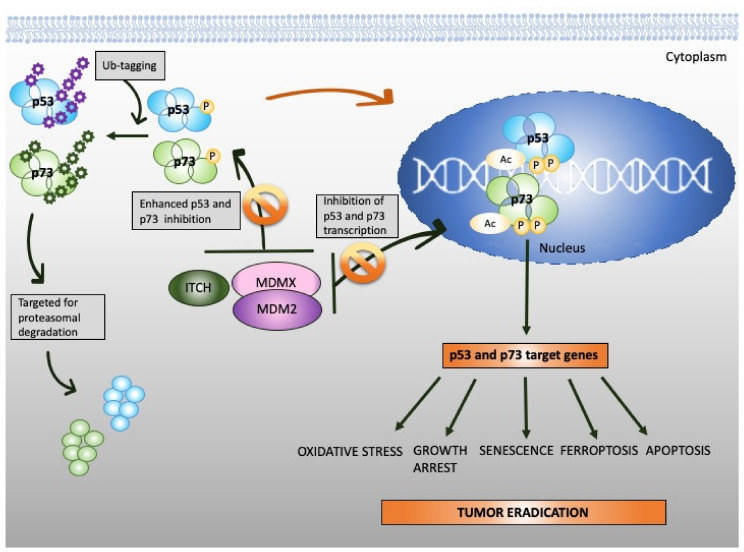
Reinstatement of p53 and p73 to treat cancer. Both p53 and p73 are rendered inactive in tumor cells through enhanced ubiquitination by MDM2/MDMX and MDM2/MDMX/ITCH axis, respectively. In addition to enhanced protein degradation, the transcriptional activity of p53 and p73 is inhibited through binding to MDM2 and MDMX. Targeting protein-protein interactions with small molecules or peptidomimetics (orange, crossed circles) stabilizes p53 and p73 and restores their transcription function (orange arrow). This, in turn, promotes tumor eradication through multiple mechanisms, as depicted in the scheme.

Structural homology between the DBD domains (Figure 2B) explains why p53 and p73 transactivate many of the same target genes, such as *PUMA*, *CDKN*1A, or *BAX*. Similarly to p53, p73 maintains the tumor suppressor function by guarding the genomic stability and driving cell cycle arrest, replicative senescence or apoptosis [78,79]. Reports also point to the involvement of p73 in regulating metabolism [80]. p73 activity is coordinated by a plethora of post-translational modifications, such as ubiquitination, phosphorylation, acetylation, or sumoylation driven by oncogenic insult or IR-mediated DNA damage [81]. Like p53, p73 transcription is inhibited by binding to MDM2 [82] and MDMX [83] through its TAD domain (Figure 3). The affinities of MDM2 and MDMX to p73 are of the same order as to p53, K_d_ (μM) = 1.4 and K_d_ (μM) = 0.22, respectively [84]. Thus, one can conclude that there is a high structural and functional similarity between the domains of p53 and p73. The similarity between the proteins was shown by molecular dynamics simulations which described similar, transient structural fluctuations of the p53 and the p73 α-helixes when in proximity to the MDM2 binding pocket [85].

p73, like p53, has both transcription-dependent and independent functions. Transcription activity of the longest form of p73, TAp73α, similarly to p53, is induced by acetylation by p300 and CREB-binding protein (CBP) acetyltransferases [86]. Next, p73 transcriptional activity and p73-driven cell death are significantly enhanced by YAP (YES-associated protein) through p300/CBP. On the other hand, YAP stability is increased by DNA damage via c-Abl kinase-mediated phosphorylation promoting the reinforced p73-mediated apoptosis. C-Abl is activated by DNA damage and is known to activate p53 [87,88]. In addition, p73 is directly phosphorylated by c-Abl at Tyr99 which further increases its transcriptional activity and enhances DNA repair driven by TAp73 [89]. In addition to promoting p73 transcription activity, YAP also outcompetes MDM2 and ITCH E3-ligase from the complex with p73, promoting TAp73 protein stability [90].

The stability of p73 is mediated by E3 ubiquitin ligase. The major E3 ubiquitin ligase of p73 is HECT ligase ITCH [91]. MDM2 and MDMX both bind to N-terminus of p73 and inhibit its transcriptional activity [92,93]. Recent studies indicated that MDM2 promotes p73 proteolytic disassembly through interacting with ITCH [94,95] and that at high levels, MDM2 polyubiquitinates p73 and regulates p73-facilitated apoptosis [95]. p73 has also cytoplasmic, transcription-independent functions and after DNA damage induces apoptosis through noncanonical binding to anti-apoptotic Bcl-XL [96].

#### 2.2.2. p73 Tumor Suppressor Function

After its discovery, the function of p73 in cancer was largely unexplored. Early studies demonstrated that the knockout of *Tp*53 leads to tumor development in mice [97]. The mice heterozygous for *Tp*73 (p73^+/−^) are tumor-prone [98], and the studies from the Tak Mak’s Lab demonstrated unequivocally that the knockout of TAp73 (TAp73^−/−^) leads to tumor development and infertility in vivo [99]. Around 70% of the mice cohort developed lung cancer, and the rest showed premature aging, which was attributed to the de-regulated metabolism. In these mice, infertility was a result of genomic instability. Aberrancy in the DNA repair system in TAp73^−/−^ mice might affect the quality of oocytes in a manner similar to the one occurring during healthy aging and thus, may explain the observed phenotype. This study demonstrated for the first time that TAp73 is a powerful tumor suppressor involved in DNA repair. Next, Elsa Flore’s Lab showed that acute genetic depletion of ΔN isoforms of p73 induced regression of tumors developed in the *Tp*53-null background in vivo [100]. The mechanism of tumor regression was via the induction of apoptosis. Altogether, deletion of ∆Np73 compensates for p53 loss and this occurs through the upregulation of TAp73 and induction of apoptosis. Another study showed that depletion of *MDM*2^−/−^ in *Tp*53^−/−^ null tumors leads to the upregulation of p73, apoptosis and tumor regression via activated p73 [101]. Thus, the above-mentioned studies and others [102] comprise a large body of evidence that demonstrates that the deregulated p73 contributes to cancer development and progression and that accumulated TAp73 compensates for p53 loss and induces tumor suppression.

#### 2.2.3. Pharmacological Reactivation of p73

Unlike *TP*53, *TP*73 gene is infrequently mutated in cancers [40,103]. Due to promoter hypomethylation, the oncogenic ΔNp73 isoform is upregulated in several cancers, including gastric, esophageal, thyroid and head and neck cancer and the cancers of the lung, breast or ovary. High ΔNp73 is linked to poor prognosis and treatment resistance [104,105]. One way to overcome oncogenic ΔNp73 is to alter the ratio between the isoforms by elevating the levels of TAp73. Whether pharmacological activation of TAp73 isoform can compensate for p53 loss has been controversial for a long time. Apart from IR-induced DNA damage, only a few molecules were described to directly or indirectly activate TAp73 in cancers [76,106]. Several pathways lead to inactivation of TAp73 in cancer. Firstly, it is the epigenetic modification at P1 and P2 which alters the ratio between TA/ΔN isoforms and next the binding to oncogenic protein inhibitors like ΔN isoforms, mutant p53 or MDM2 and MDMX. Thus, current efforts aim at direct or indirect targeting of protein–protein interactions to reactivate TAp73 in tumors.

A study using siRNA-mediated inhibition of ITCH demonstrated that cancer cells lacking p53 are more sensitive to ITCH silencing after treatment with chemotherapeutics and undergo rapid apoptosis due to p73 activation [107]. Hence, targeting ITCH-p73 interactions emerges as a promising approach for cancer therapy, which is discussed in Section 3. In addition to ITCH, p73, like p53, is subject to similar regulation by MDM2 protein. Cumulated evidence showed that at higher dose, Nutlin-3, the MDM2-p53 antagonist, induces TAp73 and apoptosis in cancer cells [108]. Furthermore, small molecule RETRA was described to target mutp53-p73 complex and to specifically suppress the growth of mutant p53-bearing tumor cells in vitro and in mouse xenografts [109]. Yet, the p73 and c-Abl kinase axis was described to significantly contribute to cisplatin-induced cytotoxicity in cancer cells with wtp53 [110]. Interestingly, another study showed that ΔNp63 mediates p73-dependent sensitivity to chemotherapy in triple-negative breast cancer [111]. Briefly, ΔNp63 promoted the survival of breast cancer cells by binding to TAp73 and inhibiting its proapoptotic activity, whereas breast cancer cells expressing ΔNp63α and high TAp73 exhibited cisplatin sensitivity that was dependent on TAp73. In response to treatment with cisplatin, TAp73 underwent c-Abl-dependent phosphorylation, which promoted dissociation of TAp73 from the complex with ΔNp63 and this triggered TAp73-dependent transcription of proapoptotic Bcl-2 family members and apoptosis. Next, a recent study showed that hypermethylation of P1 of *TP*73 gene correlates with the decrease in TAp73 and shorter overall survival of bladder cancer patients. A DNA demethylating agent, decitabine, decreased the methylation of CpGs in P1 of *TP*73 and increased the sensitivity to cisplatin in cell culture conditions [112]. The study from Christian Gaiddon’s Lab showed that HDAC significantly induces mRNA and protein levels of p73 and protein levels of p53 in gastric cancer cell lines after cisplatin treatment. This leads to the efficient induction of the proapoptotic genes *PMAIP*1 (*NOXA*) and *BIK* [113]. These findings support the key role of TAp73 in eliminating cancer cells in response to cisplatin and delineate p73 as a vital target of the drug.

In addition to the extended studies on cisplatin, p73 also sensitizes p53-null colon cancer cells (HCT 116 p53^−/−^) to withaferin A (WA), a plant-derived proteasomal inhibitor. WA stabilizes and activates TAp73 through the c-Jun N-terminal kinases - NAD(P)H dehydrogenase [quinone] 1 (JNK-NQO1) axis and reactive oxygen species-mediated response. In more detail, the study showed that WA induces p73 phosphorylation by JNK kinase, releases p73 from MDM2, stabilizes p73 on the protein level, and induces p73-dependent apoptosis in p53-null cells [114]. Next, a study with bortezomib (Velcade^®^), a known proteasomal inhibitor approved by the FDA as a frontline treatment in Relapsed/Refractory multiple myeloma, further confirmed the ‘druggability’ of p73. Here, researchers used a pair of isogenic HCT 116 human colon cancer cell lines differing only in p53 status and showed that bortezomib induces TAp73 and apoptosis in cells lacking p53 [115]. These studies too, supported the notion that p73 can be targeted with small molecules and efficiently compensates for p53 loss in tumor suppression. Thus, based on the successful reports highlighted above, the strategy aiming at the targeted restoration of TAp73 for cancer therapy is feasible, and p73 is a promising therapeutic target in cancers.

## 3. Targeting p53 Protein Family for Improved Cancer Therapy Using Repurposed Drugs

### 3.1. Drug Repurposing

The burdens of current cancer care are; systemic toxicity, and the financial toxicity of approved interventions [116]. In 2013, the experts in chronic myeloid leukemia made a strong point about the rocketing prices for cancer drugs that often do not bring a benefit to cancer patients [117]. The universal approach of pricing a new drug is primarily based on the cost of the standard care drug for the given indication plus 10–20%. The estimated cost of bringing new cancer treatment to the market was assessed to be ~USD 1 billion [118] and the recent analysis by Vinayak Prasad indicates it is USD 757 million [1]. Unfortunately, many new drugs deliver marginal benefits to the patients at a tremendous price. For example, bevacizumab, mentioned above, costs in colon cancer USD 570,000 per quality-adjusted life-year (QALY) [119] and immunotherapy up to USD 800,000 per QALY. The high costs of cancer drugs pose a serious financial burden and distress to patients due to high out-of-pocket expenses [116].

The late toxicity of current cancer treatments is linked to the higher incidence of primary cancers, including sarcoma or leukemia, later in life. A group of patients especially vulnerable to treatment-induced secondary malignancies are childhood cancer survivors [120,121]. Taking the above into account, there is a need for the establishment of less toxic and affordable treatments. One way is to apply drug repositioning. Drug repositioning uses existing drugs developed for other indications to treat other diseases including cancer [122]. A successful example of such an approach is all-trans retinoic acid (ATRA), first approved to treat acne and next successfully repurposed to cure acute promyelocytic leukemia (APL) [123].

The Anticancer Fund (AF), a not-for-profit organization, supports clinical trials on drug repurposing in cancer. AF’s recent project analyses promising, off-patent compounds as candidates for repositioning in oncology (http://www.redo-project.org/) [124]. The Repurposing Drugs in Oncology database (ReDo) lists the drugs with anti-cancer potential (310 drugs), including compounds activating p53 proteins which will be discussed in more detail in Section 3.2. [124].

### 3.2. Repurposed Drugs That Reactivate p53 and p73

When reconstituted in established tumors, p53 and p73 trigger rapid tumor regression in vivo [100,125]. The role of p53 in tumor suppression is already well-established. Importantly, despite earlier controversies regarding p73, multiple studies showed that TAp73 efficiently compensates for p53 loss in cancer and drives apoptosis after irradiation or after treatment with cisplatin or proteasomal inhibitors. Thus this, and the fact that p53 and p73 bear high structural homology, make both p53 proteins very promising, druggable targets for improved cancer therapy.

Relating to restoration of p73 by drug repurposing approach; earlier studies from Gerry Melino’s Lab, identified inhibitors of ITCH E3 ubiquitin ligase among anti-depressant drugs [126]. The compounds showed anti-cancer activity against lung cancer [127] and are speculated to restore p73 activity in cancer cells and induce p73-facilitated apoptosis. However, more detailed studies are needed to evaluate the efficacy of p73 reactivation by anti-depressant drugs in tumors.

A promising candidate for drug repurposing to reactivate p53 and p73 in cancers is protoporphyrin IX (PpIX). PpIX is a natural analog of heme, synthesized from succinyl-CoA and glycine which form aminolevulinic acid (ALA), a reaction catalyzed by ALA synthase (EC 2.3. 1.37). Ferrochelatase (FECH, EC 4.99.1.1) is an enzyme required for the incorporation of Fe^2+^ into the protoporphyrin IX ring (a heme devoid of Fe^2+^), which is the terminal step in the heme synthesis. Mutations in the human *FECH* gene, located on chromosome 18, induce protoporphyrin accumulation in the skin, erythrocytes, and liver, resulting in light sensitivity. Photosensitivity is a result of photosensitizing properties of porphyrins which absorb light at the far UV region, (Soret band) 400–410 nm and to a lesser extent in the Q bands, 580–650 nm, resulting in the generation of the excited electronic states [128]. The reduced activity of the FECH enzyme causes a rare syndrome called erythropoietic porphyria (EPP). EPP is an autosomal semi-dominantly inherited disease, manifested by the accumulation of protoporphyrin in the erythrocytes, plasma, and in hepatocytes. EPP symptoms vary depending on the type of mutation in the *FECH* gene and the degree of enzyme inhibition. The symptoms are mostly photocutaneous in the form of non-blistering lesions, however, 5% of patients progress rapidly to liver failure. In these patients, two heterozygous mutations of *FECH* ablate the enzyme’s activity and induce massive accumulation of porphyrins in the liver and consequent hepatic damage [129,130]. EPP can also be acquired, which is linked to the aberrancies in chromosome 18. A recent study showed that the EPP disease was associated with a hematological disease, largely with MDS with 18q resulting in the loss of one of *FECH* allele [131]. Surprisingly, such patients present acute symptoms with immediate painful cutaneous photosensitivity, blistering, and hepatic insufficiency. The severity of the symptoms might be explained either by the co-existing mutations in the remaining allele and further drop in FECH activity or by the aberrancies in heme synthesis pathway and iron metabolism linked to MDS which further inhibit FECH [132]. Other studies demonstrated the reduction in ferrochelatase activity in the malignant tissue by several factors when compared with that in the liver. Thus, FECH inactivation leads to enhanced accumulation of porphyrins in tumors [132,133,134]. Due to the altered activity of FECH, exogenous administration of ALA induces massive accumulation of PpIX in the diseased tissue. This phenomenon is applied in clinics to treat actinic keratosis (Table 1). So far, ALA-PpIX is used in clinics and in clinical trials together with light activation [135]. Briefly, the administration of ALA salt stimulates enhanced synthesis of PpIX and after light exposure it becomes excited. Excitation activates type 1 and/or type 2 photoreaction, leading to the generation of reactive oxygen species. ROS induces damage and cells’ eradication [136,137], which is called photodynamic therapy (PDT).

Several studies described effective repurposing of PpIX in cancer. Bednarz et al. showed that exogenous protoporphyrin IX (exo-PpIX) induced apoptotic cell death without light activation in HeLa cancer cells [138]. Exo-PpIX also promoted apoptosis in murine sarcoma cells. The mechanism of cell death was through the decrease in the mitochondrial membrane’s potential, the release and next the translocation of mitochondrial apoptosis inducing factor to the nucleus [139]. Exo-PpIX was also shown to elevate p53 and its apoptotic targets in colon cancer cells HCT 116, which induced cell death [140]. Next, Zawacka-Pankau et al. showed that p53 activation by PpIX is due to the disruption of MDM2-p53 complex. The mechanism of MDM2-p53 complex inhibition is via direct association of PpIX with N-terminus of p53 as depicted using fluorescence correlation spectroscopy. Direct binding of PpIX to p53 N-terminus was confirmed by my group, by gel filtration, anisotropy measurements, and fluorescent band shift assay [141]. Despite the advanced studies, the exact mechanism of how PpIX interacts with p53 needs to be elucidated. It is assumed that PpIX, by binding to p53, shifts the conformation of p53 α-helix containing the MDM2 binding residues and renders them unavailable for the interaction with MDM2. Detailed studies are needed to pin down which residues are responsible for the binding of PpIX to p53. Yet, further studies showed that exo-PpIX is a dual inhibitor of the MDM2-p53 and MDMX-p53 interactions, as depicted in a yeast-based reporter assay, fluorescence two-hybrid assay and immunoprecipitation [142]. PpIX induced apoptosis in leukemia cells without affecting healthy cells. Taken together, repurposed PpIX is the only compound reported to date to serve as a dual inhibitor of the p53/MDM2/MDMX interactions which binds to p53.

**Figure 4:**
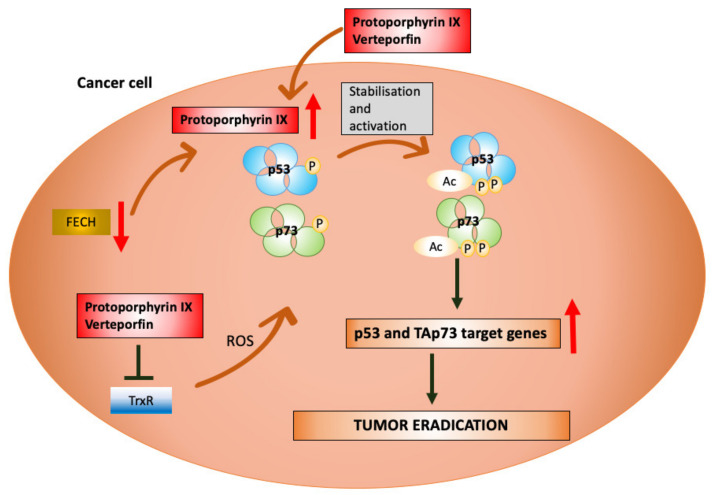
Repurposing porphyrins for improved cancer therapy. The low activity of ferrochelatase (FECH) in cancer cells leads to elevated levels of protoporphyrin IX (PpIX). High levels of PpIX sensitize malignant cells to exo-PpIX and Verteporfin, which bind to p53 and TAp73, stabilize the proteins, and induce p53- and TAp73-dependent transcriptional activity and apoptosis. Inhibition of thioredoxin reductase (TrxR) by PpIX and Verteporfin generates ROS and amplifies p53-dependent and TAp73-dependent apoptosis in cancer cells.

In addition to p53, the study using a pair of isogenic human colon cancer cell lines HCT 116, differing in p53 status, showed that cells lacking p53 are also dying of PpIX, though at different kinetics. It was reasoned that in p53-null cells, TAp73 might compensate for p53 loss and induce apoptosis. In vitro studies revealed the binding of PpIX to the p73 N-terminal domain [141]. Next, it was shown that PpIX increases p73 protein levels in p53-null cancer cells, induces TAp73-dependent transcription and activates apoptotic *NOXA* and *PUMA*. Additionally, PpIX ablated the MDM2/MDMX/p73/ITCH complex and inhibited tumor growth of p53-null subcutaneous xenografts via activation of p73 and Poly (ADP-ribose) polymerase (PARP) cleavage [143] (Figure 4).

The analog of protoporphyrin IX, benzoporphyrin derivative (BPD, verteporfin), known under the commercial name Visudyne^®^, is approved by the FDA to treat age-related macular degeneration with light activation (Table 1) and several cancer clinical trials are ongoing with Visudyne^®^ and PDT. The compound is listed in the ReDO database [124] among the compounds that are promising candidates for drug repositioning in oncology. Recent studies demonstrated that repurposed exo-PpIX and Visudyne^®^ stabilize TAp73 protein and induce apoptosis in pancreatic cancer cells with mutant *TP*53 without affecting non-transformed cells. In addition, the same study showed that both exo-PpIX and Verteporfin induce ROS and are efficient inhibitors of thioredoxin reductase (TrxR), a component of a thioredoxin-thioredoxin reductase system responsible for forming reduced disulfide bonds in cells [144]. The mechanism of p73 restoration in mutp53 cancer cells might be due to the inhibition of MDM2-p73 and the MDMX-p73 complex, as shown before in p53-null cancer cells [144]; however, this has not been yet unequivocally tested (Figure 4). Interestingly, BPD was also described to inhibit YAP-TEAD interactions and to decrease liver overgrowth in mice [145]. However, further studies are needed to fully elucidate the potential of exo-PpIX and Visudyne^®^ alone, without light activation, for improved therapy in cancers with mutp53.

FECH and ALA synthase are negatively regulated by high concentrations of heme and hemin (commercial name Panhematin^®^), an analog of heme containing the oxidized Fe^3+^ and chloride ligand (Table 1). Panhematin^®^, applied in clinics to treat acute attacks in porphyria, was recently repurposed to inhibit the metastatic spread of lung tumors in mouse model. The study demonstrated that hemin induces degradation of heme-binding transcription factor BACH1 and prevents antioxidant-facilitated metastasis in lung adenocarcinoma [146]. Interestingly, BACH1 is also a negative regulator of p53 which binds to p53 and impedes cellular senescence [147]. The binding of BACH1 to p53 is inhibited by p19ARF, like MDM2 [148]. Even though it has not been demonstrated yet, hemin might be a promising drug repurposing candidate to reactivate p53 in cancers through degradation of BACH1.

Another feasible approach to reactivate p53 protein in cancers is through induction of the ribosomal stress. Ribosomal stress upregulates RPL11 and RPL5 proteins, known negative regulators of MDM2. Chloroquine and amodiaquine, the FDA-approved anti-malaria drugs, were identified in a cell-based screen as p53-inducers [149]. A recent study repurposed amodiaquine (AQ) and showed that it stabilizes p53 through inhibition of ribosome biogenesis [150]. Amodiaquine, apart from authophagy inhibition, also inhibits rRNA transcription, a rate-limiting step for ribosome biogenesis. AQ triggered the degradation of the catalytic subunit of RNA polymerase I (Pol I) in cancer cells in the absence of DNA damage. Next, the study demonstrated that AQ stabilized p53 at low doses. The mechanism was through the inhibition of ubiquitin ligase activity of MDM2 by promoting the formation of the complex between MDM2 and RPL5/RPL11/5S rRNA. Lastly, the commercially available analog of AQ, amopyroquine (ApQ), was found to be even more effective in reactivating p53 in human osteosarcoma cells U2OS through the same mechanism [150].

Aspirin, a known anti-inflammatory drug, was first approved by the FDA as a temporary relief of minor pains and to handle fever. Recently, aspirin has been approved for the secondary prevention of stroke and acute cardiac events. Several randomized clinical trials are ongoing with aspirin as a preventive or anti-cancer agent. A randomized, double-blinded, placebo-controlled study with 325 mg aspirin in subjects with a history of colonic neoplasia showed favorable alterations in spectral biomarkers when compared with the placebo group. This study provided a procedure for the dose change/adjustment in the follow up clinical trials with aspirin as a preventive agent (ClinicalTrials.gov identifier (NCT number): NCT0046891) [151]. Several pre-clinical studies demonstrated the anti-cancer activity of aspirin in colon or gastric cancer cells [152,153]. The mechanism of tumor suppression by aspirin is not fully understood yet. Of note, aspirin was shown to acetylate wtp53 at ten lysine residues in vitro and in colon cancer cells, which led to p53 protein stabilization and accumulation in the nucleus [154]. Five out of ten lysines are a target of MDM2, which may explain nucleolar accumulation of aspirin-induced p53. Since the same study also showed that aspirin acetylates mutp53, further work is needed to elucidate the mechanism of p53 reactivation by aspirin.

Another promising example of drug repurposing in oncology is niclosamide (Table 1). It is an oral salicylanilide derivative approved by the FDA to treat intestinal tapeworm infections. The drug was shown to be able to overcome the p53 deficiency in cancer cells and manifested anti-cancer activity, specifically in mutant p53 tumors. Niclosamide is a mitochondria uncoupler and stabilizes p53 and induces p53-facilitated apoptosis. Interestingly, the cells lacking p53 were more sensitive to the drug. The study showed that the mechanism of apoptosis induction in p53-null cells was mediated by arachidonic acid and that wtp53 decreased its levels in cancer cells, rendering them less sensitive to the niclosamide [155]. It is not clear yet if p73 contributes to the observed robust induction of apoptosis in cancer cells lacking functional p53, and further studies are needed to address this matter.

Statins are the inhibitors of the 3-hydroxy-3-methylglutaryl coenzyme A reductase and interfere with sterol synthesis. The drugs are approved by the FDA as the cholesterol-lowering agents. They demonstrated anti-cancer efficacy, and showed mutp53-related response [156], however, the mechanism of action needs to be further elucidated. Interestingly, another repurposed drug, metformin, approved by the FDA to treat type II diabetes, was shown to induce p53 pathway in cancer (Table 1). Metformin inhibits mitochondrial complex 1, changes AMP/ATP ratio which induces AMP-activated protein kinase (AMPK) [157]. Under metabolic stress, such as glucose deprivation, AMPK activates p53 by phosphorylation at serine 15 [158]. Interestingly, metformin treatment inhibits MDMX by AMPK-facilitated phosphorylation at serine 342 enabling the binding of 14-3-3, which in turn decreases MDM2/MDMX ubiquitin ligase activity and stabilizes p53 [159]. In addition, other studies showed that p53 is required to induce senescence and apoptosis in breast cancer cells treated with this anti-diabetic drug [160].

## 4. Future Perspectives/Conclusions

Despite the documented success of repositioning of all-trans retinoic acid for treating acute promyelocytic leukemia, due to the persisting bias in oncology community and some critical expert views [161], many promising candidates for drug repurposing are still at the early preclinical phase (Table 1). An exception are trials with repurposed aspirin, statins, metformin, or sildenafil (Viagra^®^). In clinical trials (CTs), these repurposed drugs are applied alone, in combination with the standard care or with the metronomic chemotherapy. The trial design depends on many factors, such as the stage of the disease, previous treatments and comorbidities, available standard of care for a given indication, molecular target of the repurposed drug and the dose limiting toxicities. Up to now, there are around 170 clinical trials listed for cancer for aspirin, 180 for statins, 350 trials for metformin and 24 for sildenafil (https://clinicaltrials.gov/ct2/home). Several other promising candidates for drug repurposing in oncology, which might directly or indirectly reactivate p53 proteins, are listed in the ReDo database [124]. These drugs need to be studied further to fully understand their anti-cancer potential and cancer biology before proceeding into clinical trials. Next, in order to make the repurposed drugs an approved treatment, phase II, not-for-profit, randomized, double-blinded clinical trials with a placebo arm and patient-centered endpoints are needed on a par with well-characterized response biomarkers allowing for patient stratification.

At present, no clinical studies repurposing ALA-PpIX, exo-PpIX or Verteporfin without light activation are listed in cancer. Taking into account what we already know about the metabolism of porphyrins in cancer cells and about the mechanism of p53 and p73 reactivation by PpIX and BPD, the compounds are promising candidates for drug repurposing in oncology. Advanced studies are needed to fully apprehend the mechanism of p73 reactivation in tumors with *TP*53 mutations, still a great, unmet medical need. However, before proceeding to clinical trials with porphyrins, and other drugs discussed in the article, the studies on cancer-type specific responses and on p53 and p73 as biomarkers for patient stratification must be successfully concluded.

## Figures and Tables

**Table 1:** Promising candidates for drug repurposing in oncology targeting p53 proteins. Selected drugs were described to show a significant degree of p53-dependent action in cancer cells. All of the relevant literature is referred to in the main text. A question mark indicates a not fully depicted mechanism.

Repurposed in Cancer	Original Indication	Stage of Studies in Cancer	Mechanism
ALA-Protoporphyrin IX	Actinic keratosis (with PDT)	Pre-clinical	inhibition of MDM2-p53 and MDMX-p53; inhibition of MDM2-p73 and MDMX-p73; inhibition of thioredoxin reductase
Verteporfin	Age-related macular degeneration (with PDT)	Pre-clinical	activation of p73; inhibition of thioredoxin reductase; inhibition of YAP-TEAD
Panhematin	Porphyria	Pre-clinical	inhibition of BACH1; stabilisation of p53 (?)
Amodiaquine	Malaria infection	Pre-clinical	ribosomal stress; MDM2 inhibition
Niclosamide	Intestinal tapeworm infection	Phase I, II	mitochondrial uncoupling
Aspirin	Ache, pain, fever	Phase I–III	p53 acetylation and stabilisation
Metformin	Diabetes type II	Phase I–III	activation of p53 by AMPK-mediated phosphorylation; inhibition of MDMX by AMPK-facilitated phosphorylation
